# It is necessary to establish an International Agency for Research on Aging

**DOI:** 10.18632/aging.101451

**Published:** 2018-05-12

**Authors:** Vladimir N. Anisimov, Alexandre V. Sidorenko

**Affiliations:** 1 Department of Carcinogenesis and Oncogerontology, N.N. Petrov National Medical Research Center of Oncology, St. Petersburg 197758, Russia,; 2 European Centre for Social Welfare Policy and Research, Vienna A-1230, Austria

**Keywords:** aging prevention, pharmacological intervention, life span, preclinical trials

## Abstract

The global aging of human population is one of the main challenges and opportunities of the 21st century. Establishing an International Agency for Research on Aging as an entity affiliated to one of the intergovernmental institutions, such as the World Health Organization, can be crucial for promoting international collaboration in gerontology, in particular in a search of effective and safe geroprotectors for humans.

## Pharmacological intervention in aging as a “hot spot” of life science

Extending healthy lifespan is one of the main goals of gerontology and preventive medicine [1–5]. Recent experimental studies suggest that medications targeting aging (antioxidants, calorie restriction mimetics, autophagy inducers, etc.) can promote health and extend healthy lifespan of various animal species [2–5]. At present, more than 200 substances are listed in the Geroprotectors.org database [6,7].

There are potential interventions which might delay and/or prevent the onsets of many chronic pathologies associated with human aging [3]. The affected pathways have been identiﬁed, and the behavioral, dietary, and pharmacologic approaches to preventing and treating age-related disorders have emerged [3–5]. Interventions that target the aging process in its entirety appear to be more effective in preventing a broad range of age-related pathologies than specific interventions targeting such pathologies. Development of the new anti-aging drugs opens broad prospects for the pharmaceutical and healthcare industries [7]. However, if human longevity continues to advance, the incidences of age-associated diseases, including cardiovascular diseases, type 2 diabetes, and cancer would also increase thus presenting a tremendous challenge for humankind [1]. The search for adequate models for selecting the effective and safe methods of healthy life extension has become a priority in biology of aging.

There are at least two broadly accepted definitions of pharmacological compounds capable of intervening in aging: a) *anti-aging drugs*, which presumably reverse the aging process (‘rejuvenation’), and b) *geroprotectors,* which are supposed to prevent premature aging and/or slow down or postpone aging. Spindler [2] introduced the term “*longevity therapeutics*” for drugs that can interfere with the process of aging and extend the mean and/or maximum lifespan, preserve physiological functions and mitigate the onset and severity of a broad spectrum of age-associated diseases in mammals. Vaiserman et al. [5] have subdivided the potential geroprotective agents into several groups: those that demonstrate an anti-aging effect without any evidence of lifespan increase; those that increase lifespan by reducing the incidence of age-associated pathology; and those that extend lifespan presumably by reversing the aging process itself. Most of the evidence related to these definitions and classifications has been gained in animal studies. Laboratory animals are similar to humans in many respects, such as the patterns of aging at the molecular, cell, tissue, and physiological levels, and the responses to hazardous exposures. Nevertheless, mounting experimental evidence suggests that important differences (genetic, metabolic, ontogenetic etc.) do exist between mammalian species that hamper valid interpretations of animal experiments and their extrapolations to humans. The concordance of responses between rodent species and between rodents and humans, as well as the reproducibility and site-specificity of research findings, are important considerations in evaluating the results of experiments on laboratory animals [8,9]. How can one know whether the experimental extension of lifespan in nematodes may be applicable to humans? In the range from less to more advanced organisms, such as from nematodes through flies to mice, the magnitude of lifespan-modifying effects and their relevance to aging decline making their projections to human aging uncertain. Experiments with rapamycin are an example of this uncertainty [10,11].

## Current programs for testing interventions in aging

In 2003, the US National Institute on Aging started the Aging Interventions Testing Programme (ITP) intended to test interventions that have a potential to extend lifespan and postpone or prevent age-associated diseases and dysfunctions [12]. Among such interventions and substances are pharmacological drugs, nutriceutics, diets, plant extracts, hormones, peptides, amino acids, chelating agents, antioxidants, etc. In the framework of ITP, aspirin, nordihydroguiaretic acid, nitrofluorodiprophen, rapamycin, resveratrol and some other drugs were studied. Priority was given to the substances that are easily available, have a reasonable commercial price and can be administered preferentially with food or drinking water. The ITP protocol includes two phases. In the first phase, the ability of a drug to increase lifespan is studied. Other parameters, such as animal’s activity at various ages, metabolic hormone levels, and blood T-lymphocyte counts are also examined. In the second phase, drugs showing promising results are studied more thoroughly to reveal candidates for further clinical trials. Behavioral and cognitive experiments, assessments of the oxidative state, and postmortem pathomorphological examinations are carried out in the second phase.

Various approaches to classification of geroprotectors and investigation of their potential were recently discussed [13]. The designs of most such studies were found to have various deficiencies which led to confounding results. Therefore, there is the need to work out standard guidelines for testing such drugs and evaluating their life extending potential as well as various late effects, including tumor development. Guidelines for testing should include such significant elements as animal models, testing regimens, and biomarkers/endpoints. The procedure of the experimental preclinical study of candidate drugs should include assessing their effects on the biomarkers of aging, the lifespan, and the development of the age-associated pathologies, especially tumors. The study should be conducted in rats and mice (inbred, outbreed or genetically modified animals) treated lifelong with drugs at different doses [14,15]. The ultimate goal of such study is to select geroprotectors for studies in humans. To this end, it is necessary to develop international standards for conducting the preclinical and clinical studies of agents intended to be used in pharmacological interventions in aging, as well as for evaluating the results of such studies. In the years to come, a promising agenda could be the development of new biomarkers based mostly on biochemical and genetic tests for short-term screening of potential agents. Collaborative studies of anti-aging drugs and geroprotectors conducted in various laboratories could be particularly promising.

## International program on the evaluation of effect of geroprotectors

In 2000, an international program on the assessment of the efficacy and safety of geroprotectors was proposed [16]. It was suggested that the proposed program could be established under the auspices of the United Nations Program on Aging, the World Health Organization, and the International Association of Gerontology and Geriatrics. Unfortunately, the proposed program has not been implemented. We believe that it is worth reverting to that earlier proposal.

The aim of the proposed program is to draw up critical reviews of potential geroprotectors by a panel of international experts. The experts would suggest procedures and guidance for analyzing the activity and efficacy of geroprotective drugs and recommend additional studies, if required. Publishing the reports of the expert panel would help national and international health institutions to design and implement programs of prevention of premature aging and rehabilitation for it, as well as to make decisions about risk-benefit ratios of such programs. Experts would comment only on the evidence about the efficacy and safety of geroprotectors; they should not give direct recommendations related to the drug usage. Exclusive responsibility for the development of regulations and standards will remain with national and international health institutions.

Currently, there are no substances whose geroprotective activity has been proven in humans. However, a large body of evidence obtained in numerous animal experiments and a few clinical studies confirms geroprotective effects of metformin [17], rapamycin [4,18], melatonin [19,20], and pineal peptide preparations [21] (Table 1). These drugs seem to be the most promising candidates for testing in multicentric, randomized clinical studies.

**Table 1:** Summary of some most significant effects of promising geroprotectors in rodents.

Parameter	Metformin	Rapamycin	Melatonin	Epitalon*
Lifespan	↑	↑	↑	↑
Antioxidant capacity	↑	↑	↑	↑
Sensitivity to insulin	↑	↑	↑	↑
Low-density lipoproteins	↓	↓	↓	↓
Stress resistance	↑	↑	↑	↑
Reproductive function	↑	↑	↑	↑
Cognitive and learning performance	↑	↑	↑	↑
Physical endurance	↑	↑	↑	↑
Age-related pathology	↓	↓	↓	↓
Cancer risk	↓	↓	↓	↓

↑: increase; ↓: decrease; *Ala-Glu-Asp-Gly.

The evaluation of safety of a drug in rodents is a crucial stage of its preclinical trials. Long-term assays for carcinogenicity in rodents aim at evaluating the toxicity and other adverse effects of a tested drug [22]. Combining in one study the assessments of both the safety and the geroprotective potential of a drug could save time and significantly decrease the cost of evaluation.

## Why an International Agency for Research on Aging?

Whereas the main challenge for healthcare in the 20^th^ century had been the rapid increase in morbidity and mortality from malignant neoplasms, in the 21^st^ century the primary challenge will be the effects of global aging on public and individual health. Therefore, the establishment of an International Agency for Research on Aging (IARA) under the auspices of the World Health Organization, similar to the International Agency for Research on Cancer (IARC), is expedient. IARC, which recently celebrated its 50 anniversary [20], is a multi-disciplinary research institution bringing together experts in epidemiology, basic research and biostatistics for identifying the causes of cancer. The ultimate goal of IARC is to reduce the burden of the disease by adopting the preventive measures. A significant feature of the IARC as an independent international organization is its expertise in coordinating research across countries and organizations. The IARC has a particular interest in conducting research in low and middle-income countries through partnerships and collaborations with researchers in these regions. Since 1971, in the framework of the IARC Monographs on the Evaluation of Carcinogenic Risks to Humans, working groups of expert have critically reviewed the published studies and evaluated the weight of evidence on more than 900 agents. Starting in 1995, the IARC Handbooks of Cancer Prevention series have provided evaluations of the cancer-preventive potential of numerous agents and interventions. IARC plays a key role in establishing cancer registries around the world and in monitoring cancer burden worldwide and its geographical variations and trends over time. Education and training programs are also among central aspects of IARC activity. Initially founded by six countries in 1965, IARC memberships has grown as of today up to 25 countries. Over 50 years IARC has shaped the scope of cancer research [23].

Similarly to IARC, the objective of IARA should be the promotion of international collaboration in gerontology and geriatrics. The main activities of IARA could be focused on the following:

➢ scoring the demographic aging on the five continents;

➢ coordinating the international projects on genetics of aging and longevity for identification of gender, ethnic, geographic and socio-economic determinants of longevity;

➢ developing international program for verification and unification of biomarkers of aging;

➢ establishing international guidelines for the interventions to prevent premature aging;

➢ unifying protocols for the preclinical studies of potential geroprotectors;

➢ performing meta-analysis of the clinical trials of geroprotectors in humans in accordance with the principles of evidence-based medicine;

➢ organizing training programs for researchers and practitioners and educational programs for general public.

In the long run, IARA could also become a coordinating and reference center for social and economic studies of aging thus providing indispensable evidence for designing, implementing and monitoring national and international policies on aging, including evidence for supporting the implementation of the main global policy framework on aging – the Madrid International Plan of Action on Aging [24].

Vladimir Anisimov has more than 40 years of experience in testing drugs for their potential to influence the aging process, lifespan, and carcinogenicity, as well as 35 years of experience of collaborating with WHO, IARC and International Programme of Chemical Safety. The co-author, Alexandre Sidorenko, for 20 years had led the UN Programme on Ageing. We both are confident that it is the international collaboration that would make it possible to find a right path in the still less travelled world of anti-aging research.
